# Changes in Rice and Livestock Production and the Potential Emergence of Japanese Encephalitis in Africa

**DOI:** 10.3390/pathogens10030294

**Published:** 2021-03-04

**Authors:** Jennifer S. Lord

**Affiliations:** Department of Vector Biology, Liverpool School of Tropical Medicine, Liverpool L3 5QA, UK; jennifer.suzanne.lord@gmail.com; Tel.: +44-0151-705-3146

**Keywords:** Japanese encephalitis virus, Africa, anthropogenic change

## Abstract

The known distribution of Japanese encephalitis (JE) is limited to Asia and Australasia. However, autochthonous transmission of Japanese encephalitis virus was reported in Africa for the first time in 2016. Reasons for the current geographic restriction of JE and the circumstances that may permit emergence in non-endemic areas are not well known. Here, I assess potential changes in vector breeding habitat and livestock production in Africa that are conducive to JEV transmission, using open-source data available from the Food and Agriculture Organization between 1961 and 2019. For 16 of 57 countries in Africa, there was evidence of existing, or an increase in, conditions potentially suitable for JE emergence. This comprised the area used for rice production and the predicted proportion of blood meals on pigs. Angola, where autochthonous transmission was reported, was one of these 16 countries. Studies to better quantify the role of alternative hosts, including domestic birds in transmission in endemic regions, would help to determine the potential for emergence elsewhere. In Africa, surveillance programs for arboviruses should not rule out the possibility of Japanese encephalitis virus (JEV) circulation in areas with high pig or bird density coincident with Culicine breeding habitats.

## 1. Introduction

Japanese encephalitis virus (JEV) caused >100,000 cases of Japanese encephalitis (JE) and *c*. 25,000 deaths in 2015 [1]. There are 24 JE endemic countries in Asia, with sporadic outbreaks in Australasia [1,2]. Existing evidence suggests that the geographic distribution of JE is limited to these two regions; however, isolations of JEV have recently been made in Africa and Europe [3,4,5]. In Africa, JEV RNA was isolated from a human presenting with yellow fever symptoms during the 2016 yellow fever outbreak in Angola [4]. 

Japanese encephalitis virus is mosquito borne and maintained in transmission cycles involving pigs and/ or birds [6]. The presence of JE in Asia has been associated with rural areas supporting both rice paddies and pig farming [6,7,8,9]. Rice paddies support suitable habitat for larvae of *Culex* vector species, particularly *Culex tritaeniorhynchus* [10]. However, recent studies in Asia have shown JEV circulation in peri-urban areas and isolation from peri-urban mosquitoes [11,12]. 

Despite records of JEV in new regions and alternative ecological contexts, relatively little research has been done to better understand the limiting factors for JEV maintenance and spread, or the potential for emergence in new regions, including Africa and Europe (summarised in [13,14], but see [15]). *Culex tritaeniorhynchus* does occur in at least 19 countries in Africa [16], and other competent vector species are likely present.

The potential for JEV invasion and onward transmission will depend on factors at scales smaller than national level, including relative numbers of competent and dead-end hosts and the presence of suitable vector habitat. Vectors, including *Cx. tritaeniorhynchus*, considered most important for JEV transmission in Asia, have a preference for feeding on cattle relative to pigs [14,17]. Cattle, however, do not produce sufficient viremia to infect mosquitoes and are therefore dead-end hosts for JEV [18]. Amplifying transmission of JEV can only occur if sufficient blood meals are taken from pigs or other competent hosts [7]. The transmission rate of a mosquito-borne virus is particularly sensitive to changes in the proportion of blood meals on competent hosts because this parameter is involved in both the force of infection from host-to-mosquito as well as mosquito-to-host. Therefore, host community composition in addition to competent host density is likely an important factor in determining the potential for JEV invasion and subsequent transmission in a given context.

Quan et al. [1] noted that the estimated force of infection for JEV from animals to humans broadly correlated with pig density and *Cx. tritaeniorhynchus* probability of presence across Asia (as estimated by [19,20]). Sub-national data on livestock and potentially suitable vector habitat to inform the potential for JE emergence is not, however, widely available. The Food and Agriculture Organization (FAO) provide open-source records, from 1961 to present day, for crop and livestock production at a national level [21]. These data may be useful for monitoring changes in livestock and rice production to indicate regions of changing JEV risk. While this approach has a clear set of limitations, which I outline in the Discussion, it serves as a starting point to draw attention to changing ecological contexts at a broad scale, which may help to target resources to studies at scales relevant to transmission.

I therefore aimed to (i) first quantify the potential effect of the proportion of blood meals on competent hosts on the basic reproduction number for JEV; (ii) estimate predicted vector blood meals on pigs using FAO pig and cattle density data and estimates of vector host preference [17], for JE endemic countries in 2015 (to coincide with estimates of the force of infection by Quan et. al. [1]) and for African countries over time; and (iii) compare land mass dedicated to rice production for the JE endemic countries in 2015 with that for African countries over time. Collectively, these data inform hypotheses about why JE may not have emerged previously in Africa, but also highlight the potential for emergence in some countries due to increases in pig production relative to cattle, coincident with increases in rice production.

## 2. Results

**Figure 1:**
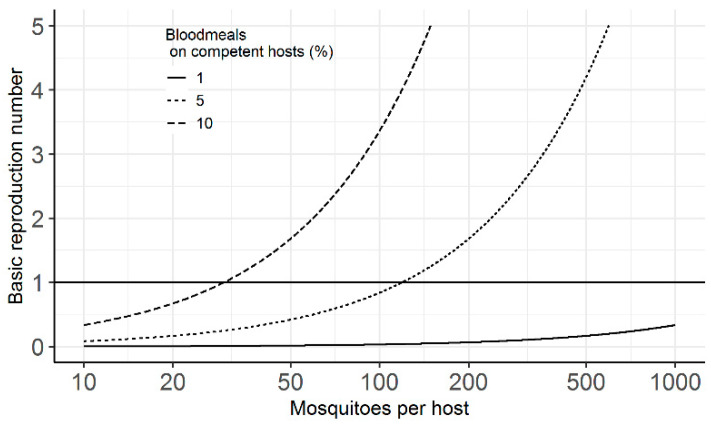
Basic reproduction number (R_0_) for a mosquito-borne pathogen according to variable blood meals on competent hosts (%) and the mosquito: host ratio. Other parameters held constant: biting rate—1/3 days^−1^, extrinsic incubation period—7 days, mosquito-to-host transmission probability—1, host-to-mosquito transmission probability—1, mosquito survival rate—0.9 days^−1^ and host recovery rate—1/5 days^−1^.

I used an equation for the basic reproduction number (R_0_) of a mosquito-borne pathogen [22], adapted to include the proportion of blood meals on pigs (*σ*), to relate blood feeding to the potential for JEV to invade. I show that even with a short extrinsic incubation period of seven days, a biting rate of 1/3 days^−1^, and transmission probabilities equal to one, *c*. 5% of blood meals would have to be on pigs, in the absence of other competent hosts, for an R_0_ > 1, assuming the average mosquito: host ratio is *c*. 100:1 (Figure 1).

**Figure 2:**
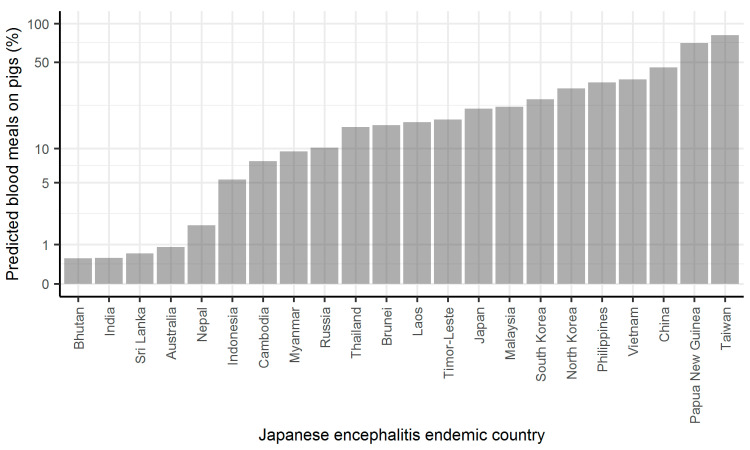
Predicted percentage of blood meals on pigs for Japanese encephalitis endemic countries. Estimates were calculated using the host feeding index (see Methods) using data on country-level pig and cattle density in 2015 [21] and host preference of *Cx. tritaeniorhynchus* for cattle (0.9) and pigs (0.1) [17]. Note *y* axis is on a log scale.

Given the effect of the proportion of blood meals from competent hosts on R_0_ (Figure 1), it was anticipated that if national-level data were somewhat indicative of JE risk, the majority of JE endemic countries would have a predicted percentage of blood meals on pigs ≥5%, based on estimates of vector preference [17] and relative density of pigs and cattle from FAO data [21]. Indeed, this was the case for estimates using FAO data from 2015, with the predicted percentage of blood meals on pigs ≥5% for 77% of 22 JE endemic countries (Figure 2). No pig data were available for Bangladesh, Pakistan or Singapore, but, at least for Bangladesh, pigs are present in some regions, albeit in relatively low density [14,23]. There were five other countries in Asia with low predicted percentage of blood meals on pigs. These observations are considered in the Discussion. 

**Figure 3:**
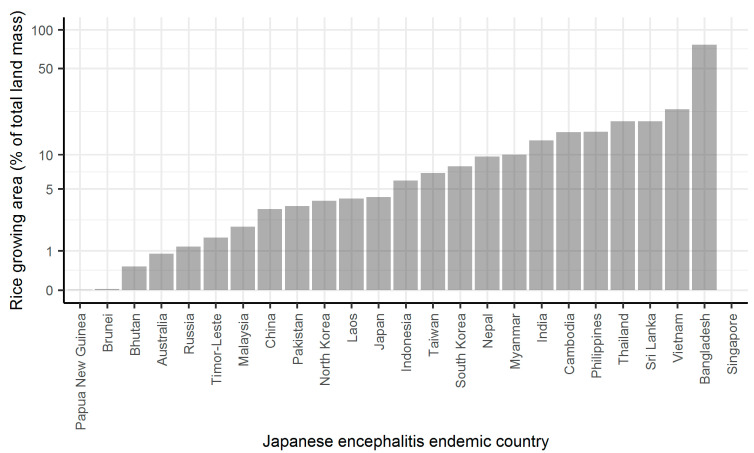
Percentage of land mass used for growing rice in 2015 for Japanese encephalitis endemic countries. Data from Food and Agriculture Organization (FAO) [21]. Note *y* axis is on a log scale.

Next, I calculated rice paddy area as a percentage of total land mass for each of the JE endemic countries, using data on harvested area from FAO and the total area of each country (Figure 3). National level estimates of percentage of area harvested ranged from <1% to >75%, but for 87% of 25 countries at least 1% of land mass was used for rice production.

**Figure 4:**
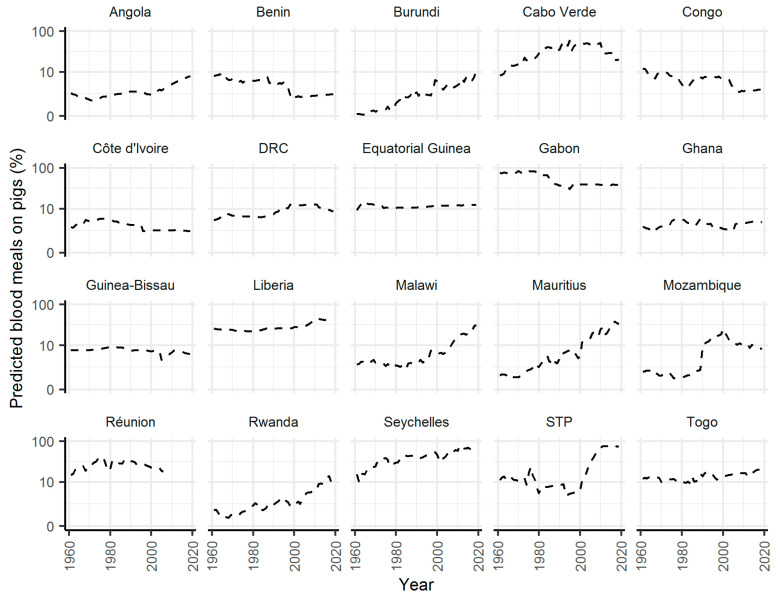
Changes in the predicted percentage of blood meals on pigs for countries in Africa. Only countries with at least one year with the predicted value ≥5% are shown. Calculated as per Figure 2. Data used to produce the figure from FAO [21]. DRC—The Democratic Republic of Congo, STP—Sao Tome and Principe.

As a means of assessing changes in the potential suitability of African countries for JEV over time, I estimated the predicted percentage of blood meals on pigs, in addition to rice paddy area between 1961 and 2019 for 57 African countries. Thirty five percent of 57 countries had a predicted percentage of blood meals on pigs ≥5% for at least one year between 1961 and 2019. Angola was one of these countries, with the predicted percentage of blood meals on pigs increasing from 2.4% in 1961 to 7.6% in 2019 (Figure 4). Of the 20 countries, just six (10% of all countries) have consistently had a predicted percentage of blood meals on pigs ≥5% over this period. In addition to Angola, predictions have increased in Burundi, Malawi, Mauritius, Mozambique, Rwanda, and Sao Tome and Principe since the 1960s (Figure 4).

**Figure 5:**
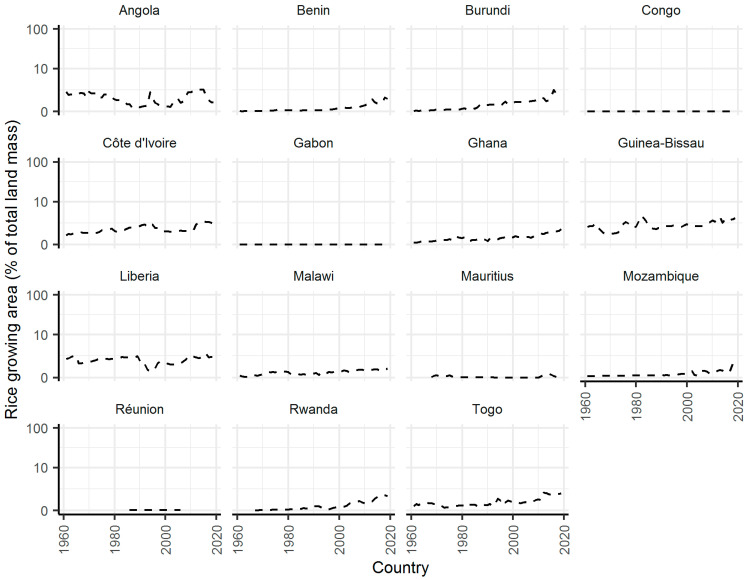
Percentage of total land mass used for rice growing in African countries between 1961 and 2019. Only countries with predicted percentage of blood meals on pigs ≥5% for at least one year shown. Data used to produce the figure from FAO [21].

For the 20 countries shown in Figure 4, I subsequently estimated the area of land mass used for rice production. Of these countries, four had no data, effectively indicating no recorded rice production. The data for the remaining 16 countries are shown in Figure 5. Of note is the relatively small area used for rice production in comparison with countries in Asia. Of these 16 countries, 10 had ≥1% of land mass used for rice for at least one year. Four countries—Angola, Côte d’Ivoire, Guinea-Bissau and Liberia—had >30 years where rice producing area was ≥1%. However, for eight countries rice production has increased since the 1960s, particularly since 2000 (Figure 5). 

## 3. Discussion

The occurrence of JE has long been associated with intensification of rice production and rural pig farming in Asia [6]. Here, I have provided evidence in support of the hypothesis that the ecological context in most African countries has been unsuitable for JEV amplifying transmission, which requires sufficient densities of vectors and susceptible, competent hosts. Regarding the detection of JEV in a human in Angola during the 2016 yellow fever virus outbreak [4], the virus could have been recently introduced by migratory birds or imported mosquitoes or could already have been maintained at low levels in wild birds for some time. 

Whether those countries that may support suitable conditions for JEV invasion and onward transmission also support potential routes of entry for JEV remains to be quantified. The most obvious way JEV could be introduced to a new region is through bird migration. Indeed, this was likely the reason for identification of JEV in birds and mosquitoes from Italy [3]. The countries in Africa that may be suitable for supporting JEV transmission could be compared with data available on bird migratory routes between Asia and Africa but were beyond the scope of this initial study. 

Angola was one of the countries that had both rice production covering ≥1% of the land mass and increasing pig production relative to cattle. Of the other countries on mainland Africa, those in west Africa—Côte d’Ivoire, Guinea-Bissau and Liberia—were among the few that may have supported sufficient pig and rice production in previous decades that could have led to amplifying transmission of JEV. In addition, there are recent increases in both pig farming and rice production in other countries of sub-Saharan Africa, including Malawi, Mozambique and Rwanda. 

There are, of course, limitations to the approach that should be acknowledged when drawing conclusions. It was difficult to decide a reasonable range for the ratio of mosquitoes to hosts, and in regions of JEV transmission, this may well be higher than the range used in this analysis, but likely only sustained for a limited number of months per year (e.g., [24,25]). I focus only on pigs as competent hosts and provide reasoning for this in the Methods. While there are data to indicate domestic birds may be important to transmission [26,27], there are few studies that have quantified the necessary parameters to properly implicate domestic birds or assess their relative importance to transmission in contexts with and without pigs. This has already been raised as a gap in JE research [14]. Indeed, there were countries in JE endemic regions that supported relatively low numbers of pigs and low percentage of land mass for rice production. It may be that in these regions duck farming contributes to JEV transmission risk. I have also not considered the role of wild birds, with the assumption that the density of wild hosts is infrequently sufficient for amplifying transmission to levels of risk to humans. Japanese encephalitis epidemics in Asia are usually associated with pig farming (summarised in [28]). This assumption will likely not hold in all situations, and the possibility of targeted analyses where there are wild bird data available would be useful. Indeed, West Nile virus is maintained across Africa in a transmission cycle involving wild birds and mosquitoes, including vector species which have been shown to also be competent for JEV [29,30]. 

While I also only focus on rice paddy as a source of vector larval habitats, there is some evidence that *Cx. pipiens* and other vectors adapted to stagnant or small bodies of standing water may be able to transmit the virus in peri-urban environments [31]. Again, further research on the role of this species in this context is warranted. The conclusions are also dependent on the assumption that the host feeding preferences used to estimate the proportion of blood meals on pigs is the same for African vectors as for vectors in Asia, but this could well be different and other vector species could also be competent for infection and subsequent virus transmission.

Climate and climate change was not considered in this assessment, but recent work indicates that, at least for *Aedes aegypti*-borne arboviruses, the area with suitable climate is expected to expand from west Africa throughout sub-Saharan Africa [32]. 

Lastly, and most importantly, conclusions drawn from national-level data must be taken with caution because drivers of transmission will vary at a smaller scale. The relative arrangement of vector breeding habitats, competent hosts and dead-end hosts will ultimately determine the risk for invasion and subsequent amplifying transmission. Further work on whether and how force of infection varies at the sub-national level due to variation in vector breeding habitat and host community composition in regions atypical of the traditional transmission context is warranted to help inform more quantitative estimates of risk of spread to new geographic areas. More generally, studies in endemic regions to compare mosquito prevalence of infection, or force of infection from mosquitoes to livestock against livestock census and rice production data, would be useful to quantify whether, and at what scale, these could be suitable indicators of risk. 

Despite the above limitations, this study serves to bring attention to potential for changes in rice and livestock production in some regions of Africa that may permit amplifying transmission of JEV. Countries in sub-Saharan Africa, including Angola [33], plan to increase rice production in coming years (e.g., https://riceforafrica.net/ accessed on 28 February 2021). Further research on Culicine mosquitoes in rice and other crop production systems in Africa would enable informed decision making about how to reduce the risk of arbovirus emergence under expanding crop production systems. Aside from the first recording of autochthonous transmission in Angola, I am not aware of any other studies on the risk of emergence of JE in Africa.

## 4. Materials and Methods

To show the effect of the proportion of vector blood meals on competent hosts, on the potential for JEV invasion, I used the following equation for the basic reproduction number (R_0_) for a mosquito-borne pathogen [22], adapted to include the proportion of bites on competent hosts (*σ*):

(1) $$R_{0}=\;\frac{{\left(\sigma a\right)}^{2}bcp^{n}m}{r\left(-\ln p\right)}\;$$

with the following fixed parameters: *a*—mosquito biting rate = 1/3 days^−1^; *b*—probability of transmission from mosquito to host = 1; *c*—probability of transmission from host to mosquito = 1; *p*—mosquito survival rate = 0.9 days^−1^; *n*—extrinsic incubation period = 7 days (see S2 file of [30]); and *r*—host recovery rate = 1/5 days^−1^ [34]. Number of mosquitoes per host (*m*) was varied between 10 and 1000. This was used to illustrate the potential effects of variation in host community composition—particularly competent vs. dead-end hosts, in combination with vector host preference, on the potential for JEV invasion.

Following this, JE endemic and African countries were compared using the parameter rho, which was calculated as

(2) $$\sigma =\frac{f_{P}N_{P}}{\left(f_{P}N_{P}+f_{C}N_{C}\right)}\;$$

where *f_P_* is the feeding preference of *Cx. tritaeniorhynchus* (the main vector in Asia and also present in Africa) on pigs is 0.1 and *f_C_* on cattle is 0.9 [17]. *N_P_* and *Nc* are numbers of pigs and cattle, respectively, obtained from the FAO database [21]. The focus on pigs and cattle only is based on: (i) the innate host preference for primary JEV vectors for cattle and the absence of similar data for domestic birds from field experiments; and (ii) the absence of large-scale duck farming in the majority of African countries, as established based on FAO data. Of the few studies that focus on the potential contribution of domestic birds, Ladreyt [27] estimated the force of infection on ducks as 0.03 per month and chickens as 0.003. While chickens produce viremia sufficient to infect mosquitoes [26], there is not convincing evidence that sufficient blood meals on chickens occur to support transmission. In the Discussion, I discuss this limitation with respect to observations both for JE endemic countries and for African countries.

The above approach was used to estimate the predicted proportion of blood meals on pigs (*σ*) for the countries, which form 30 endemic regions as described by Quan et. al. [1]. Livestock census data for 2015 were downloaded from the FAO database [21]. This year was chosen as it is the year for which force of infection estimates are available for each region [1]. Countries without pig data were omitted, but these are highlighted in the results. The resulting estimates are reported as a percentage. For the same set of countries, I estimate the proportion of land mass used for rice production, again using data from the FAO database on the area harvested for rice paddy and dividing this by the total land mass for each country. 

For African countries, the area of land occupied by rice and the number of livestock, between 1961 and 2019 were downloaded from the FAO database. The same calculations for the predicted proportion of blood meals on pigs and the proportion of land mass used for rice production as calculated for JE endemic regions were utilized. 

## Data Availability

Data was obtained from the FAO and are available from the FAO at http://www.fao.org/faostat/en/#data/QC (accessed on 28 February 2021), under the Open Data Licensing Policy (http://www.fao.org/contact-us/terms/en/, accessed on 28 February 2021).
